# Nutrition Knowledge, Food Insecurity, and Dietary Biomarkers: Examining Fruit and Vegetable Intake Among College Students

**DOI:** 10.3390/nu17030584

**Published:** 2025-02-05

**Authors:** Emily Sklar, Marcela D. Radtke, Francene M. Steinberg, Valentina Medici, Deborah S. Fetter, Rachel E. Scherr

**Affiliations:** 1 Department of Nutrition, University of California, Davis, CA 95616, USA; essklar@ucdavis.edu (E.S.); fmsteinberg@ucdavis.edu (F.M.S.); dsfetter@ucdavis.edu (D.S.F.); 2 Department of Epidemiology and Population Health, Stanford School of Medicine, Stanford University, Palo Alto, CA 94304, USA; mdradtke@stanford.edu; 3 Department of Internal Medicine, Division of Gastroenterology and Hepatology, University of California, Davis, CA 95616, USA; vmedici@ucdavis.edu; 4 Family, Interiors, Nutrition & Apparel Department, San Francisco State University, 1600 Holloway Avenue, San Francisco, CA 94132, USA; 5 Scherr Nutrition Science Consulting, San Francisco, CA 94115, USA

**Keywords:** food insecurity, college students, dietary assessment, nutrition knowledge, carotenoids, fruit and vegetable intake

## Abstract

Objectives: Food insecurity among college students, combined with limited nutrition knowledge and barriers to healthy eating, significantly impacts diet quality and fruit and vegetable intake. Efforts to address these issues are further complicated by the challenges of accurately and efficiently collecting dietary data in research settings. This study aimed to explore the relationship between nutrition knowledge and fruit/vegetable intake using skin, plasma, and dietary carotenoid levels as biomarkers. Methods: Undergraduate and graduate students aged 18 years and older (*n* = 166) from a California public university were recruited. The sample was predominately female (*n* = 133, 80%), with 30 males (18%) and three individuals (2%) identifying as non-binary. Food security was assessed using the USDA’s 10-item Adult Food Security Survey Module and nutrition knowledge through a validated questionnaire. Biological data included blood samples and skin carotenoid measurements (Veggie Meter^®^). Dietary quality (HEI-2015) and carotenoid intake were assessed through Diet ID™, a photo-based assessment tool. Results: The mean nutrition knowledge scores were 36.55 ± 8.83 out of 58 points, and the mean skin carotenoid score was 307.07 ± 110.22. Higher knowledge scores were associated with increased plasma carotenoids, HEI-score, and Diet ID™ total carotenoids. Food security classification did not significantly impact nutrition knowledge but did influence HEI scores and skin carotenoid levels, with very low food security linked to poorer diet quality and lower carotenoid levels. Conclusions: Nutrition knowledge may serve as a significant predictor of fruit and vegetable intake in university students. Despite this correlation, the impact of overall diet quality is potentially hindered by an individual’s food security status. Therefore, while knowledge is critical, addressing food insecurity is essential for enhancing diet quality among college students.

## 1. Introduction

College students represent a diverse subgroup of the adult population, who experience increased financial stressors, such as high living costs and tuition expenses, as well as reduced work capacity due to competing academic priorities. Collectively, these contribute to the disproportionate rate of food insecurity in this population [1,2]. Food insecurity, defined as a condition of limited or uncertain access to adequate food, emerges as a fundamental determinant of physical and emotional well-being [3].

In addition to financial stressors, college students encounter a multitude of barriers that profoundly impact their dietary choices, which include limited nutrition knowledge, cooking skills, time constraints, and restricted access to food and transportation, among others [4,5]. Often, attending college marks the first time that students are responsible for their own dietary habits and food acquisition [1]. Poor dietary habits among students are characterized by increased consumption of processed and ultra-processed foods, fast foods, calorically-dense snacks, sweets, sugar-sweetened drinks, alcoholic beverages, and low or inadequate intake of fruits and vegetables (F/V) [6,7]. Results from the National College Health Risk Behavior Survey demonstrate that among college students, 74.4% do not meet the Dietary Guidelines for Americans recommendation of at least five servings of F/V a day [8]. Inadequate consumption of F/V poses a critical concern for college students, considering the important health benefits associated with achieving dietary recommendations for these foods [9,10,11]. Fruits and vegetables contain antioxidants and serve as sources of essential vitamins and minerals, including some nutrients of public health concern (calcium, potassium, and fiber) [9,10,12]. Adequate consumption of F/V has been consistently linked to improved physical health and body composition, and reduced risk of many chronic diseases, including cardiovascular disease, certain cancers, and type II diabetes [9,11,12,13,14]. Fruit and vegetable consumption has also been linked to better academic and educational outcomes; college students with higher diet quality and greater intake of F/V exhibit higher grade point averages and decreased drop-out rates, as compared to those with poorer dietary quality and lower F/V intake [15,16,17].

Variations in diet quality and F/V consumption in the college student population can be influenced by a range of factors, including gender, race/ethnicity, socioeconomic background, student standing, housing, and nutrition knowledge [18,19,20,21,22,23,24]. Research across populations, including children, college students, and adults, supports the positive association between increased nutrition knowledge and improved dietary behaviors [25,26]. Exploration into the barriers and enablers of healthy dietary intake underscores the role of nutrition knowledge in fostering healthy eating behaviors, particularly regarding food choices and meal composition [5]. In parallel with the relationship between nutrition education and dietary intake, positive associations between improved nutrition knowledge and food security status have been observed among college students [27,28,29,30,31,32,33]. Students with higher levels of nutrition knowledge are more likely to be food secure than students with lower nutrition knowledge [34,35]. Greater nutrition knowledge scores may reflect the knowledge of food access resources, foods that support healthy dietary habits, and elevated cooking self-efficacy [36,37,38]. Although there is evidence supporting the benefits of nutrition education in collegiate learning environments, there is no universal mandatory nutrition requirement that must be met across universities. Interventions aimed at improving nutrition knowledge have been postulated to be part of an effective strategy for positively influencing students’ dietary behaviors and food security status in the college student population [5,32].

Gaining a comprehensive understanding of the relationship between nutrition knowledge and dietary behaviors, specifically F/V intake, requires a rigorous collection of dietary intake data. There is a wide selection of dietary assessment tools used to capture dietary intake and F/V consumption; however, each tool poses a distinct set of challenges and limitations that can introduce measurement error and biases [39]. Collecting accurate dietary data can be challenging, burdensome, and tedious for both researchers and research participants [39,40]. Among the most frequently utilized methods for capturing dietary intake are the food frequency questionnaire (FFQ) and 24-h dietary recalls [39]. It is crucial to acknowledge that both the FFQ and 24-h dietary recalls are susceptible to random and systematic errors given by the episodic nature of food choices and the participants’ capacity to recall food consumption accurately [39]. Although online or app-based food recall methods have been developed to improve the accuracy of dietary data collection and alleviate some of this burden, they do not completely eliminate the challenges associated with subjective bias and recall error [41,42]. Similarly, clinical or objective dietary measures, such as blood biomarkers or tissue biopsies, may help address issues related to response bias and error, but they are often resource-intensive and costly to perform [43]. The utilization of innovative technology, including spectroscopy as a proxy for F/V consumption, offers a non-invasive and cost-effective alternative to assess dietary intake [44,45].

Carotenoids are lipophilic compounds synthesized in plants, responsible for the yellow, orange, and red pigments in a wide variety of fruits and vegetables. As the human body cannot synthesize carotenoids, they must be obtained through the diet, making them a proxy for fruit and vegetable consumption. Carotenoid levels can be measured in the blood, tissue, and skin, serving as an objective indicator of dietary intake. However, as previously mentioned, sample collection and analysis can often be time-consuming and burdensome for participants. The Veggie Meter^®^ is a validated, research-grade instrument that uses pressure-mediated reflection spectroscopy to estimate carotenoid concentration in the skin, which reflects 30 days of F/V intake [45,46,47,48]. The Veggie Meter^®^ provides a non-invasive, quick, and reliable method to assess and promote healthy dietary habits and increase F/V consumption. While previous studies have examined the relationship between nutrition knowledge and fruit and vegetable consumption, they have been limited by reliance on recall-based dietary assessments [39,41]. This study is novel in two key ways: first, it utilizes the Veggie Meter^®^ to measure fruit and vegetable intake, reducing the limitations of self-reported recall data; second, it explores the correlation between nutrition knowledge biomarkers and overall diet quality, providing a more comprehensive understanding of dietary behavior.

This study aims to elucidate the relationship between nutrition knowledge and F/V intake, as well as their relationship with food security status, using carotenoids as a biomarker of interest measured through both traditional and innovative assessment methods. Thus, the objective of this study is to investigate if nutrition knowledge is associated with carotenoid levels in the skin, plasma, and diet. It is hypothesized that students with greater nutrition knowledge will have higher carotenoids, which is indicative of increased F/V intake and improved diet quality, than students with lower nutrition knowledge scores.

## 2. Materials and Methods

The protocol and procedures for this study were approved by the University of California, Davis (UC Davis) Institutional Review Board. Participants provided informed written consent prior to study commencement (1476178-4). Data were collected as part of a larger study. Inclusion criteria consisted of participants currently enrolled at UC Davis, over the age of 18, and within a BMI range of 18.5–34.9 kg/m^2^ [49,50]. Individuals who smoked or resided with indoor smokers (including cigarettes, e-cigarettes, vaping, or marijuana), those who consumed THC-containing edible products, or indulge in excessive alcohol intake (>5 drinks per week) were excluded as the impact of these behaviors on the metabolism and absorption of carotenoid compounds remains uncertain [51]. Further, those who engaged in artificial tanning methods or took a high-dose Vitamin A medication orally or topically (e.g., Accutane, retinol cream) were ineligible due to the potential interference with carotenoid detection in the skin from sources other than diet [52].

A detailed description of the study procedures and sample size calculations can be found elsewhere [53]. Briefly, a convenience sample of undergraduate and graduate students attending UC Davis were recruited through flyers, classroom announcements, and email distribution lists. Prospective participants were screened and those deemed eligible were invited to participate in the study and attend an in-person clinical visit. Recruitment started in January 2020, and data were collected until the end of the academic quarter (March 2020).

Recruitment and data collection took place over two distinct periods of time. Cohort 1 occurred from January 2020 to March 2020 (*n* = 42). Following the completion of Cohort 1, the COVID-19 pandemic necessitated an immediate pause in data collection efforts. Given the clinical nature of the research and the well-being of both participants and research personnel, it was paramount to suspend data collection until conditions improved and permitted for a safe continuation (March 2022). This choice was motivated by the researchers’ acknowledgment of the seriousness of the health crisis and the necessity to follow protocols designed to reduce the risk of COVID-19 transmission. In March 2022, recruitment and data collection recommenced for Cohort 2 and carried on until the end of the term in June 2022 (*n* = 132).

### 2.1. Anthropometric Data

Anthropometric data were collected by trained researchers at a single time point in accordance with the Anthropometry Procedures Manual (2021) [54]. Height and weight underwent two independent measurements each to ensure accuracy within 0.3 cm and 0.1 kg, respectively, and the average value was recorded. Height was assessed with a stadiometer, while weight was measured using a digital scale. Subsequently, BMI was computed by dividing weight in kilograms by the square of height in meters (kg/m^2^).

### 2.2. Sociodemographic Data

Sociodemographic information, including age, gender, race/ethnicity, food security status, first-generation student status, and weekly physical activity duration, were collected. Food security status was measured using the 10-item USDA Adult Food Security Survey Module (AFSSM), which measures food security using a series of behaviors and conditions, such as anxiety about running out of food, reduced quality and quantity of food consumption, and frequency of skipping meals [55]. Using the USDA coding scale, participants were classified on a four-range scale: High Food Security (0 affirmative responses), Marginal Food Security (1–2 affirmative responses), Low Food Security (3–5 affirmative responses), or Very Low Food Security (6–10 affirmative responses) [56]. Additional questions on food access resource use were included.

### 2.3. Nutrition Knowledge Questionnaire

Nutrition knowledge was assessed using a questionnaire that had previously been validated and used in adult and college student populations [57]. The Nutrition Knowledge Questionnaire comprises 60 questions derived from several previously validated questionnaires [58,59,60], notably the General Nutrition Knowledge Questionnaire [61], and then subsequently modified with input from the Dietary Guidelines for Americans [62] and MyPlate recommendations [63]. Questions were centered around three primary themes: MyPlate [63], disease–diet relationships, and nutrient composition of food. Using the devised scoring method, scores for the Nutrition Knowledge Questionnaire ranged from 0 to 58.

### 2.4. Reflection Spectroscopy

Skin carotenoids were measured using the Veggie Meter^®^. Following the Veggie Meter^®^ standardized protocol for data collection, triplicate measurements were taken, and an average was calculated [44]. The Veggie Meter^®^ was calibrated before use and every subsequent hour of use. The non-dominant ring finger was used to mitigate inter- and intra-individual variability, as well as environmental interferences [44].

### 2.5. Diet ID

Participants completed the Diet ID™ dietary assessment at the in-person visit [53]. Diet ID™ is a validated tool that assesses dietary patterns through the use of a patented image-based algorithm known as Diet Quality Photo Navigation (DQPN) [53]. Diet ID™ works by presenting composite images of a variety of foods through a “this or that” approach and instructs the user to choose which food images are most representative of their own diet. This process continues until the best possible fit is achieved, taking approximately five minutes in duration [64]. Once Diet ID™ identifies the user’s usual eating pattern, a final image undergoes nutrient analysis using the Nutrition Data System for Research (NDSR) database to evaluate diet quality based on the Healthy Eating Index 2015 (HEI-2015) criteria [65]. Diet ID™ nutrient intakes have demonstrated a significant correlation with 24-h dietary recalls, FFQ, skin and plasma carotenoids, and blood markers [53,64,66,67].

### 2.6. Plasma Carotenoids

Fasting blood samples were obtained at the in-person visit via venipuncture by a skilled phlebotomist at the UC Davis Ragle Human Nutrition Research Center using light-protected EDTA Vacutainer collection tubes. Carotenoid levels were assessed in the plasma using high performance liquid chromatography (HPLC), following the established protocols outlined by Craft [68,69]. Additional details on plasma carotenoid analysis are described in detail elsewhere [53].

### 2.7. Statistical Analysis

Given the unanticipated expanded duration between cohorts, chi-squared and t-test analyses were performed to identify any differences between the two cohorts. With no significant cohort differences observed, data from Cohort 1 and Cohort 2 were analyzed collectively. Data were inspected for normality using Shapiro–Wilks and log transformed, when necessary. Participant characteristics are expressed as absolute (*n*) and relative frequencies (%) for categorical variables, and continuous variables are described as mean +/− standard deviation (SD).

Associations between nutrition knowledge, diet quality, and carotenoid concentrations in the plasma, skin, and diet were explored using Pearson and Kendall’s Tau correlations. To further explore the associations between nutrition knowledge, diet quality, and carotenoids, univariate and multivariable linear regression models were constructed, controlling for self-identified gender, race/ethnicity, first-generation student status, food security status, and BMI in the adjusted models. ANOVA, with Tukey’s post hoc comparisons, were performed to understand the impact of food security status on nutrition knowledge scores and markers of dietary intake, as college students are particularly vulnerable to experiencing food insecurity. Analyses were performed using STATA v13 (StataCorp, College Station, TX, USA), and statistical significance was considered with *p* < 0.05.

## 3. Results

### 3.1. Participant Demographics

A total of 166 participants finished the study with complete data and were included in the analysis. Participant characteristics, including gender, race/ethnicity, first-generation student status, food security status, and BMI, are presented in Table 1. Participants primarily identified as female (80%) and the ethnic/racial classification of participants were as follows: African American/Black (1%), American Indian/Alaskan Native (0%), Asian/Pacific Islander (56%), White not of Hispanic origin (33%), Chicano/Hispanic/Latinx (23%), and other (3%). Moreover, 40% of participants identified as first-generation college students, indicating that neither parent completed a 4-year college degree [70]. Within the population, 56% of individuals were classified as having high food security, 21% with marginal food security, 15% with low food security, and 8% with very low food security (Table 1). Participants had a mean BMI of 23.53 ± 4.53 kg/m^2^, fitting within the healthy BMI classification range (18.5 to 24.9). Additionally, participants exhibited a mean nutrition knowledge score of 36.55 ± 8.83 and a mean skin carotenoid score of 307.07 ± 110.22 (Table 1).

Nutrition knowledge scores were compared with plasma carotenoids, skin carotenoids, and dietary intake data, including dietary carotenoids and diet quality, to understand the relationship between knowledge and F/V intake.

### 3.2. Nutrition Knowledge and Diet ID

Nutrition knowledge scores were significantly associated with diet quality measured in accordance with the HEI-2015 (r = 0.17, *p* = 0.03), as assessed by Diet ID™ (Table 2). Significant associations between nutrition knowledge and the dietary intake of total carotenoids and α-carotene were observed (r = 0.17, *p* = 0.03, and r = 0.20; *p* = 0.01, respectively), with the exception of β-carotene and lutein/zeaxanthin, which were approaching significance (r = 0.15, *p* = 0.05, and r = 0.14; *p* = 0.06, respectively). After adjusting for self-identified gender, race/ethnicity, first-generation student status, food security status, and BMI, higher nutrition knowledge scores were associated with an increase in HEI-score (0.21 (0.00, 0.41), *p* < 0.05) and an increase in Diet ID total carotenoids (2.26 (0.26, 4.26), *p* = 0.03) (Table 3).

### 3.3. Nutrition Knowledge and Plasma Carotenoids

Higher nutrition knowledge scores were associated with increased plasma carotenoid levels (Table 2; *p* < 0.01 for total and individual carotenoids). Plasma total carotenoid scores, α-carotene, β-carotene, and lutein/zeaxanthin were found to be significantly correlated with nutrition knowledge scores, both within univariate and adjusted models, as shown in Table 4 (*p* < 0.001 for both models).

### 3.4. Nutrition Knowledge and Skin Carotenoid Scores

Although approaching significance, there was no significant association observed between skin carotenoid scores and nutrition knowledge scores (Table 2; r = 0.14; *p* = 0.07), likely due to the large variation in Veggie Meter^®^ scores (307.07 ± 110.22). As the detection of skin carotenoid scores is impacted by adiposity, BMI was significantly inversely associated with skin carotenoid scores (*p* < 0.001) in the adjusted model (Table 3).

### 3.5. Nutrition Knowledge, Diet Quality, and Skin Carotenoids by Food Security Status

Given the high prevalence of food insecurity in the college student population, exploring the role of food security status on nutrition knowledge and biomarkers of interest was warranted (Table 4). There was no significant difference in nutrition knowledge scores (*p =* 0.39), Diet ID carotenoid scores (*p* = 0.39), or plasma carotenoids (*p* = 0.16) by food security status. Significant differences in HEI scores by food security status were observed, such that students with very low food security had lower overall diet quality (*p* = 0.04) and lower skin carotenoid scores (*p* = 0.03).

## 4. Discussion

The primary objective of this study was to investigate the relationship between nutrition knowledge and dietary intake, as measured by HEI-scores and carotenoid levels in the blood, skin, and diet. While previous literature highlights the potential benefits of nutrition education and the relationship between nutrition knowledge on diet quality and F/V consumption, limited research has been conducted in a clinical setting using objective biomarkers within the college student population [25,26,38]. Within the present study, findings suggest that higher nutrition knowledge scores are associated with higher diet quality, as depicted by increased HEI-scores and F/V intake estimated by Diet ID and plasma carotenoid levels.

Within the study population, higher nutrition knowledge scores were correlated with increased HEI diet quality scores, total carotenoids, and a-carotene, as measured by Diet ID™. These findings are consistent with other analyses that demonstrated a positive relationship between increased nutrition knowledge and improvements in total diet quality, indicative of increased F/V intake, lower saturated fat, and reduced sugar-sweetened beverage intake, as measured through dietary assessment tools, such as the Dietary Instrument for Nutrition Education, and FFQ [32,71,72]. Poor diet quality has been previously linked to increased chronic disease risk and excess body fat [73,74,75]. The findings from this study reinforce that improved nutrition knowledge may support nutrition-related behavior change, consistent with models for behavior change like the Social Cognitive Theory [76]. The findings of this study expand upon previous research identifying nutrition knowledge as a mediator of F/V consumption within college students’ health and reduce their risk for diet-related chronic diseases [36,37].

Greater nutrition knowledge was also associated with increased plasma alpha-carotene, β-carotene, and lutein and zeaxanthin levels. Plasma carotenoid levels serve as a biomarker of F/V intake and are reflective of approximately 7–10 days of dietary intake [77]. Plasma carotenoids have been previously validated to correlate with total F/V intake measured through various dietary intake assessments, such as FFQs and 24-h dietary recalls, as well as image-based tools, such as Diet ID [53,78,79,80]. Plasma carotenoid scores have been previously associated with decreased chronic disease risk, such as cardiovascular disease and several types of cancers, due to the ability of carotenoids to serve as antioxidants, in addition to their antiapoptotic and anti-inflammatory factors [81,82,83,84,85,86,87]. As evidenced by these results, by improving nutrition knowledge, college students may be more likely to make healthy dietary choices, which can contribute to better overall health.

Despite an association between diet and plasma carotenoids with nutrition knowledge, the relationship observed between nutrition knowledge and skin carotenoid scores only approached significance. While the mean carotenoid score of 307 falls within previously identified ranges in college students, this study found that BMI, rather than nutrition knowledge, was a stronger predictor of skin carotenoid levels in the college student population [53,88]. The current study observed an inverse relationship between skin carotenoid scores and BMI, such that a higher BMI was associated with lower skin carotenoids, which is consistent with other studies regarding the use of the Veggie Meter^®^ and other spectroscopy-based skin carotenoid measures [48,89]. While BMI is not a direct measure of body composition [90], higher adiposity may contribute to lower detectable carotenoid levels in the skin due to the lipophilic nature of carotenoids and their storage in adipose tissue [52,91,92,93]. Additionally, higher BMIs are associated with oxidative stress, inflammation, and metabolic changes that further disrupt carotenoid levels in the skin, which highlights the complex interplay between body composition and nutrient biomarkers [81,89,94].

While increasing nutrition knowledge serves as a critical strategy to improve diet quality across college students, it is not the only factor that influences dietary intake. When stratifying across food security status, students with very low food security had poorer diet quality and lower carotenoid levels; however, there were no significant differences in nutrition knowledge across all food security status classifications. This indicates that while students across different levels of food security may have similar knowledge about nutrition, their ability to apply this knowledge to improve diet quality and overcome food security-related barriers, such as finances or accessibility, may vary significantly. Given that food insecurity is a public health crisis affecting 66% of California college students [95], exploration into student dietary behaviors has identified a variety of food acquisition practices and coping strategies to maintain or improve their food security [96]. These behaviors can include purchasing inexpensive groceries, which can be less nutritious, and skipping meals or strategically rationing food when supplies are low, supported by the significantly lower diet quality and carotenoid levels in students with very low food security [97,98,99]. While this study did not specifically explore coping strategies, it can be postulated that these coping mechanisms can provide a temporary solution for students; they can also lead to poor diet quality and disordered eating behaviors [97]. Moreover, food insecurity has been previously associated with lower diet quality, including reduced fruit and vegetable consumption and higher intakes of fast foods, sugar-sweetened beverages, and added sugar [100,101,102]. Thus, these findings suggest that while nutrition knowledge can support students in making more informed dietary choices, it may be insufficient to overcome barriers and challenges presented by food insecurity within students.

## 5. Limitations

The reliance on cross-sectional data and self-reporting methods presents a limitation, as these approaches can introduce bias and may not accurately capture long-term dietary patterns. A limitation of this study is the overrepresentation of female participants. Further, given the diverse racial and ethnic backgrounds of the college student population at UC Davis, some commonly consumed culturally relevant foods might not have been represented in the images shown in Diet ID™. To address this, the Diet ID™ algorithm is being enhanced to include a more extensive database of culturally diverse foods. This expansion aims to better encompass the variety of eating patterns in the US and to identify dietary trends in other regions globally.

## 6. Conclusions

The findings from this study highlight that nutrition knowledge can serve as a predictor of fruit and vegetable consumption among college students. Despite this correlation, the impact on overall diet quality is hindered by challenges, such as food insecurity and related barriers. Therefore, while nutrition knowledge is critical, addressing broader systemic issues related to food access, and nutrition security is essential for enhancing dietary habits among college students.

## Figures and Tables

**Table 1:** Participant characteristics expressed as number and percentages for self-identified gender, race/ethnicity, food security status, and first-generation student status and mean ± standard deviation for BMI, carotenoid scores, and nutrition knowledge scores.

Self-Identified Gender	
Male	30 (18%)
Female	133 (80%)
Non-Binary	3 (2%)
Race/Ethnicity	
African American/Black, not of Hispanic origin	2 (1%)
American Indian/Alaska Native	0 (0%)
Asian/Pacific Islander	90 (54%)
White not of Hispanic origin	33 (20%)
Chicano/Latin/Hispanic (Mexican–American, Puerto Rican, Cuban)	36 (22%)
Other	5 (3%)
Food Security Status	
High	93 (56%)
Marginal	34 (21%)
Low	25 (15%)
Very Low	14 (8%)
First-Generation Student Status	
Yes	67 (40%)
No	98 (59%)
Unsure	1 (1%)
BMI (mean ± SD; kg/m^2^)	
Total	23.52 ± 4.53
Male	24.76 ± 4.10
Female	23.19 ± 4.57
Non-binary	25.73 ± 5.67
Skin Carotenoid Scores (mean ± SD)	307.07 ± 110.22
Nutrition Knowledge Scores (mean ± SD)	36.55 ± 8.83
Diet ID Carotenoid Scores (mean ± SD)	29,354.73 ± 17,274.15 mcg
Plasma Carotenoid Scores (mean ± SD)	156.65 ± 74.79 mcg/dL
HEI Scores (mean ± SD)	72.95 ± 21.06

**Table 2:** Correlation coefficients between nutrition knowledge scores, diet quality, and carotenoid intake predicted by Diet ID^TM^, plasma carotenoid levels, and skin carotenoid levels measured by the Veggie Meter^®^.

Outcome Variables	Correlation Coefficient	*p*-Value
Diet ID		
HEI Score	0.17	0.03
Total Carotenoids	0.17	0.03
α-Carotene	0.20	0.01
β-Carotene	0.15	0.05
Lutein and Zeaxanthin	0.14	0.06
Veggie Meter^®^		
Skin Carotenoid Scores	0.14	0.07
Plasma		
Total Carotenoids	0.31	<0.001
α-Carotene	0.33	<0.001
β-Carotene	0.28	<0.001
Lutein and Zeaxanthin	0.21	<0.01

**Table 3:** Univariate and multivariable linear regression models exploring the relationship between nutrition knowledge scores, HEI scores and carotenoids from Diet ID, plasma, and skin.

Carotenoids	Model 1: Univariate		Model 2: Multivariable Linear Regression Model *	
	β-Coefficient (95% CI)	*p*-Value	β-Coefficient (95% CI)	*p*-Value
Diet ID HEI-Score	0.23 [0.03, 0.43]	0.03	0.21 [0.00, 0.41]	<0.05
Diet ID Carotenoids	2.24 [0.29, 4.18]	0.02	2.26 [0.26, 4.26]	0.03
Skin Carotenoids	0.18 [−0.02, 0.38]	0.07	0.12 [−0.07, 0.31]	0.22
Plasma Carotenoids	2.10 [1.12, 3.08]	<0.001	1.85 [0.88, 2.82]	<0.001

* Multivariable linear regression models were adjusted for self-identified gender, race/ethnicity, first-generation student status, food security status, and BMI.

**Table 4:** Differences in nutrition knowledge scores, diet quality, and carotenoid scores by food security status (FSS) analyzed by ANOVA with Tukey’s post hoc corrections (*n* = 166).

	High Food Security (*n* = 93)	Marginal Food Security (*n* = 34)	Low Food Security (*n* = 25)	Very Low Food Security (*n* = 14)	*p*-Value
Nutrition Knowledge	37.42 ± 9.49	35.35 ± 9.37	36.56 ± 6.34	33.64 ± 6.08	0.39
HEI Scores	73.18 ± 20.68 ^a,b^	79.03 ± 18.59 ^a^	71.04 ± 21.32 ^a.b^	60.07 ± 24.54 ^b^	0.04
Diet ID Carotenoid Scores	29,758.02 ± 18,670.05	31,814.63 ± 15,276.1	26,995.12 ± 14,969.23	29,415.24 ± 17,085.16	0.39
Veggie Meter^®^	314.48 ± 116.38 ^a^	307.97 ± 108.20 ^a,b^	320.16 ± 95.87 ^a^	232.21 ± 69.96 ^b^	0.03
Plasma Carotenoids	163.89 ± 82.92	159.84 ± 74.9	149.29 ± 51.22	114.03 ± 27.82	0.16

Values are listed as means ± standard deviation. Values with different letters in a row are significantly different (*p* < 0.05) based on ANOVA analysis with Tukey’s post hoc corrections by food security status.

## Data Availability

Data, analytical code, and research materials described in this study will be made available from the corresponding author upon request, pending application and approval.
